# Unlocking RNA mysteries: Predicting subcellular localizations with AI

**DOI:** 10.1016/j.omtn.2025.102481

**Published:** 2025-02-21

**Authors:** Nguyen Quoc Khanh Le

**Affiliations:** 1 In-Service Master Program in Artificial Intelligence in Medicine, College of Medicine, Taipei Medical University, Taipei 11031, Taiwan; 2 AIBioMed Research Group, Taipei Medical University, Taipei 110, Taiwan; 3 Translational Imaging Research Center, Taipei Medical University Hospital, Taipei 110, Taiwan

## Main text

Long non-coding RNAs (lncRNAs) have emerged as pivotal regulators of cellular processes, with their subcellular localization offering critical insights into their functions.^1^ Aberrant lncRNA localization has been linked to a range of diseases, from cancers to neurodegenerative disorders, underscoring the importance of understanding their distribution within cellular compartments. Traditional methods, such as fluorescence *in situ* hybridization (FISH), are resource intensive and not scalable for high-throughput studies. Addressing this challenge, Li et al. introduce LncDNN, a machine learning model tailored to predict lncRNA localization within the nucleolus and nucleoplasm, offering a transformative approach to RNA biology.^2^

**Figure 1:**
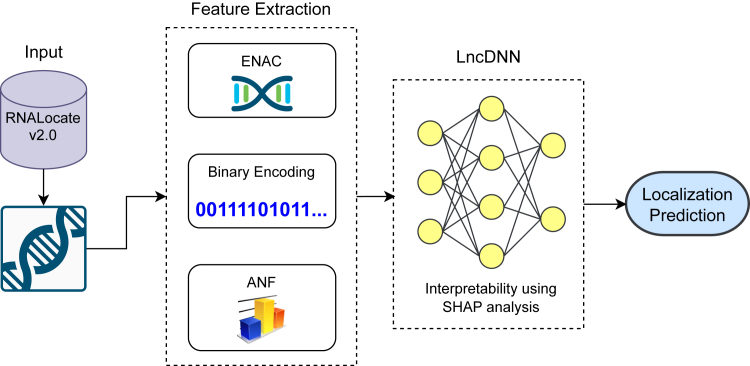
Overview of lncRNA localization prediction using LncDNN ENAC, enhanced nucleic acid composition; ANF, accumulated nucleotide frequency; SHAP, Shapley additive explanations.

Their findings, published in *Molecular Therapy Nucleic Acids*,^2^ showcase the potential of LncDNN to revolutionize our understanding of lncRNA biology. By integrating diverse feature sets and leveraging Shapley additive explanations (SHAP) interpretability analysis (Figure 1), the model achieved exceptional predictive performance, with AUCs of 0.873 and 0.831 on validation and test datasets, respectively. Beyond prediction, the study sheds light on the sequence features influencing localization, opening avenues for experimental exploration.

The complexity of lncRNA biology lies not only in their sequence diversity but also in their spatial dynamics within cells. Localization patterns often dictate lncRNA function, influencing processes such as transcriptional regulation, RNA stability, and protein interactions. In diseases like cancer, mislocalized lncRNAs can act as oncogenes or tumor suppressors, making them attractive targets for therapeutic intervention. Yet, the scalability of traditional methods limits their utility in large-scale studies. The advent of computational approaches, especially those employing machine learning, has transformed genomics and transcriptomics research. These tools offer a scalable, cost-effective means to analyze vast datasets, predict functional attributes, and infer biological mechanisms. LncDNN exemplifies this paradigm shift, providing both predictive accuracy and biological interpretability.

Li et al. adopted a data-driven approach, harnessing the RNALocate v.2.0 database^3^ to curate a high-quality dataset of lncRNA sequences localized to the nucleolus and nucleoplasm. To enhance model generalizability, sequences were clustered at 90% similarity, ensuring unique samples for training and testing. The model’s architecture incorporated three complementary feature sets.

(1) Enhanced nucleic acid composition (ENAC): captures global sequence composition patterns.
(2) Binary encoding: encodes positional information of nucleotides.
(3) Accumulated nucleotide frequency (ANF): reflects cumulative sequence insights.

The integration of these features enabled LncDNN to capture intricate sequence patterns influencing localization. Importantly, the use of SHAP analysis provided interpretability, identifying key sequence motifs and positional features driving predictions. This interpretability bridges the gap between computational predictions and experimental validation, fostering confidence among biologists.

The predictive capabilities of LncDNN extend beyond academic curiosity. Accurate localization predictions could aid in identifying lncRNAs as biomarkers or therapeutic targets. For instance, nucleolar-localized lncRNAs often influence ribosome biogenesis and cellular stress responses, pathways frequently dysregulated in cancer.^4^ Similarly, nucleoplasmic lncRNAs can modulate transcriptional networks, with implications for developmental disorders and neurodegeneration.^5^ Moreover, the interpretability of LncDNN addresses a long-standing challenge in AI-driven biology: understanding the “why” behind predictions. By identifying sequence features linked to localization, the model provides testable hypotheses, enabling targeted experimental validation and functional studies.

While LncDNN represents a significant advancement, certain limitations warrant discussion. The reliance on RNALocate v.2.0,^3^ while comprehensive, may introduce biases inherent to the database. Expanding the dataset to include diverse cell types, species, and experimental conditions would enhance model robustness and generalizability. Another limitation lies in the lack of experimental validation of the model’s predictions. Future studies could integrate *in vitro* and *in vivo* assays to confirm the localization and functional roles of predicted lncRNAs. Additionally, extending the model to predict localization across more subcellular compartments or dynamic cellular states could further broaden its applicability.

Li et al.’s work exemplifies the potential of machine learning in RNA biology, offering a roadmap for future innovations.^2^ By combining predictive accuracy with biological interpretability, LncDNN sets a benchmark for computational tools in genomics. The integration of such models with multi-omics data could unlock deeper insights into RNA biology, revealing novel therapeutic targets and biomarkers.

As computational tools evolve, their synergy with experimental workflows will be crucial. Collaborative efforts between computational scientists and experimental biologists can bridge the gap between prediction and validation, accelerating discoveries in RNA biology. The journey to fully understand lncRNA localization and function is far from over, but LncDNN represents a significant step forward, illuminating new paths for exploration and innovation.

## Acknowledgments

This work is supported by the National Science and Technology Council, Taiwan (grant number MOST111-2628-E-038-002-MY3).

## Declaration of interests

The author declares no competing interests.
